# Hepatosplenic Protective Actions of *Spirulina platensis* and Matcha Green Tea Against *Schistosoma mansoni* Infection in Mice via Antioxidative and Anti-inflammatory Mechanisms

**DOI:** 10.3389/fvets.2021.650531

**Published:** 2021-04-30

**Authors:** Amany M. Ramez, Ehab Kotb Elmahallawy, Gehad E. Elshopakey, Amira A. Saleh, Samar M. Moustafa, Ashraf Al-Brakati, Walied Abdo, Dina M. M. El-Shewehy

**Affiliations:** ^1^Zoology Department, Faculty of Science, Mansoura University, Mansoura, Egypt; ^2^Department of Zoonoses, Faculty of Veterinary Medicine, Sohag University, Sohag, Egypt; ^3^Department of Clinical Pathology, Faculty of Veterinary Medicine, Mansoura University, Mansoura, Egypt; ^4^Department of Medical Parasitology, Faculty of Medicine, Zagazig University, Zagazig, Egypt; ^5^Department of Zoonses, Faculty of Veterinary Medicine, Benha University, Benha, Egypt; ^6^Department of Human Anatomy, College of Medicine, Taif University, Taif, Saudi Arabia; ^7^Department of Pathology, Faculty of Veterinary Medicine, Kafrelsheikh University, Kafr El-Sheikh, Egypt; ^8^Zoology Department, Faculty of Science, Mansoura University, Mansoura, Egypt

**Keywords:** *Spirulina platensis*, matcha green tea, *Schistosoma mansoni*, oxidative stress, inflammation

## Abstract

Schistosomiasis, a major parasitic illness, has high morbidity and negative financial effects in subtropical and tropical countries, including Egypt. The present study investigated the therapeutic effects of *Spirulina platensis* (SP) and matcha green tea (MGT) in *Schistosoma mansoni-*infected mice combined with tracing their possible antioxidant and anti-inflammatory impacts and their protective potency. A total of 60 Swiss albino mice were randomly allocated into six groups (*n* = 10): control group (CNT, received normal saline); SP–MGT group [received oral SP (3 g/kg bodyweight/day) plus MGT (3 g/kg bodyweight/day)]; *S. mansoni* group (infected with *S. mansoni* cercariae, 100 ± 10/mouse, using the tail immersion method); SP-infected group (infected with *S. mansoni* and received oral SP); MGT-infected group (received oral MGT after *S. mansoni* infection); and SP–MGT-infected group (received combined treatment of SP and MGT after *S. mansoni* infection). Treatment with SP and MGT started 4 weeks after *S. mansoni* infection and ended 10 weeks after. SP and MGT treatment (SP-infected and MGT-infected groups) and the combined treatment (SP–MGT-infected group) minimized the hepatic damage induced by *S. mansoni*; circulating alanine aminotransferase and aspartate transaminase decreased, and total protein, albumin, and globulin serum levels increased. The serum level of malondialdehyde significantly declined, and catalase, glutathione peroxidase, superoxide dismutase, and total antioxidant capacity increased in SP-infected, MGT-infected, and SP–MGT-infected groups compared with the infected group. Co-administration of SP and MGT reduced serum cytokine levels (tumor necrosis factor-alpha, interferon-gamma, and interleukin-13) and increased interleukin-10 levels after *S. mansoni* infection compared with the infected group. Moreover, treatment with SP and/or MGT decreased the number of granulomas in hepatic and splenic tissues compared with the infected group. Collectively, our results suggest that combined SP and MGT treatment is effective for *S. mansoni* infection. Liver and spleen tissue alterations were improved, the antioxidant systems were stimulated, and the inflammatory response was suppressed. Further research is recommended to investigate the mechanisms of the combined SP and MGT treatment effects to facilitate the development of novel therapies against this disease.

## Introduction

Schistosomiasis is a debilitating waterborne disease caused by helminthic parasites belonging to the genus *Schistosoma* (1). Despite vigorous control efforts, it is still the most widespread tropical disease. Schistosomiasis, which has high morbidity, affects ~210 million people in ~76 countries, in addition to malaria and tuberculosis (2). Importantly, in Sub-Saharan Africa, 200,000 cases or more of schistosomiasis are reported annually (3). One of the causative agents of schistosomiasis is *Schistosoma mansoni*, which migrate from the blood to the lungs and liver and finally reside as male and female worms in the mesenteric veins. During the chronic phase of the disease, intense granulomatous lesions develop in the intestine, liver, lungs, brain, spleen, and pelvic organs (4). Drug-resistant *Schistosoma* strains have emerged in endemic areas due to the continuous and irregular use of the same chemotherapy against the disease (5). Thus, the development of new, dynamic, and safe antischistosomal drugs, particularly from natural plant extracts (6), is imperative.

Of note, a natural antioxidant and herbal medications are being evaluated. Recently, increased attention has been paid to the use of herbal medications as remedies for various parasitic diseases (7–10). *Spirulina platensis* (SP) is a natural spiral-shaped, multicellular photosynthetic, blue-green microalga (*Cyanobacterium*). It is commonly used as a nutritional supplement for both humans and animals (11, 12). SP has a high nutritional value; it is rich in carbohydrates (15–25%), proteins (55–70%), sterols, and polyunsaturated fatty acids (18%) (13, 14). SP contains phycocyanin, mixed carotenoids, phytonutrients, essential amino acids, gamma-linolenic acid, linoleic acid, and palmitic acid, which can improve the defense system and induce potent scavenging activities to reactive oxygen species (15, 16). Moreover, the regular consumption of SP can improve the hematological profile owing to the presence of calcium, iron, copper, magnesium, and several vitamins (B6 and B12 and folic acid) essential for hemopoiesis (17). As a food additive, SP has antitumor, antibacterial, antiviral, anticancer, anti-HIV, and anti-inflammatory activities (18–20).

Green tea (*Camellia sinensis*) is consumed worldwide, especially in Japan and China (21). Polyphenolic catechins, including epicatechin gallate, epicatechin, epigallocatechin, and epigallocatechin gallate, are the most abundant biologically active green tea components. These components have a potentially wide range of therapeutic benefits, including anti-inflammatory, antioxidant, anticancer, antidiabetic, anticholesterol, anti-obesity, anti-mutation, anthelminthic, and antimicrobial effects (22–25). The protection of tea leaves from sunlight increases their amino acid content, particularly theanine, which has a lower catechin content (26). Matcha is a finely powdered green tea manufactured from tea leaves cultivated under shade for almost 3 weeks before being harvested (27). In recent years, the consumption of matcha has increased instead of green tea. However, research on the protective effects of matcha is scarce. In addition, high caffeine levels were detected in matcha; the young leaves and buds of camellia plants have more caffeine than mature leaves (28). Given the information mentioned earlier, matcha is considered the best-grade green tea; it is rich in theanine and caffeine but little catechin contents, unlike the popular green tea (29). To the best of our knowledge, there is little data regarding the antioxidant and anti-inflammatory properties of matcha and SP against *S. mansoni* infection. Therefore, the present study was conducted to evaluate the therapeutic effects of SP and matcha green tea (MGT) on *S. mansoni* infection. The antioxidant and anti-inflammatory impacts of SP and matcha and their ability to protect the hepatic and splenic tissues after *S. mansoni* infection were investigated.

## Materials and Methods

### Ethical Considerations

Ethical approval and protocol approval for this study were obtained from the Animal Ethics Committee of the Faculty of Veterinary Medicine, Kafrelsheikh University, Egypt. The study protocol complied with all the relevant Egyptian legislation concerning the publication and research. The ethical approval number is KFS-2019/2.

### Algae and Plant Material

The SP algae were kindly supplied by the Department of Botany, Faculty of Science, Mansoura University (Mansoura, Egypt). The MGT powder was provided by Aiya Co., Ltd. (Aichi, Japan). The matcha consisted mainly of polyphenol (10.0%), caffeine (3.0%), fiber (39.0%), protein (31.0%), calcium (0.42%), potassium (2.7%), iron (0.02%), and vitamins (C, 0.06%; A, 0.005%; and carotene, 0.029%).

### Parasite Material and Preparation

Schistosoma Biological Supply Program at Theodor Bilharz Research Institute (Giza, Egypt) provided *Biomphalaria alexandrina* snails that shed *S. mansoni cercariae*. Briefly, the infected snails were kept for approximately 4 weeks in a test tube containing distilled water and then exposed to artificial light at 28°C ± 1 for 2 h to induce shedding of cercariae. The number of cercariae was then counted under a microscope, and each animal was infected by a 1-h exposure of the tail to a suspension containing 100 ± 10 cercariae (86).

### Experimental Animals and Ethical Statement

Male Swiss albino mice (*n* = 60), weighing 20 ± 25 g, were purchased from the Schistosome Biological Supply Program unit at Theodor Bilharz Research Institute (Giza, Egypt). Before administering the different treatments, the mice were quarantined in plastic cages for 2 weeks and provided with water and control diet *ad libitum*. During the experimental period, all animals were strictly handled under standard environmental conditions (12-h light/dark cycles; relative humidity, 60%; room temperature, 22 ± 2°C).

### Experimental Protocol

**Figure 1:**
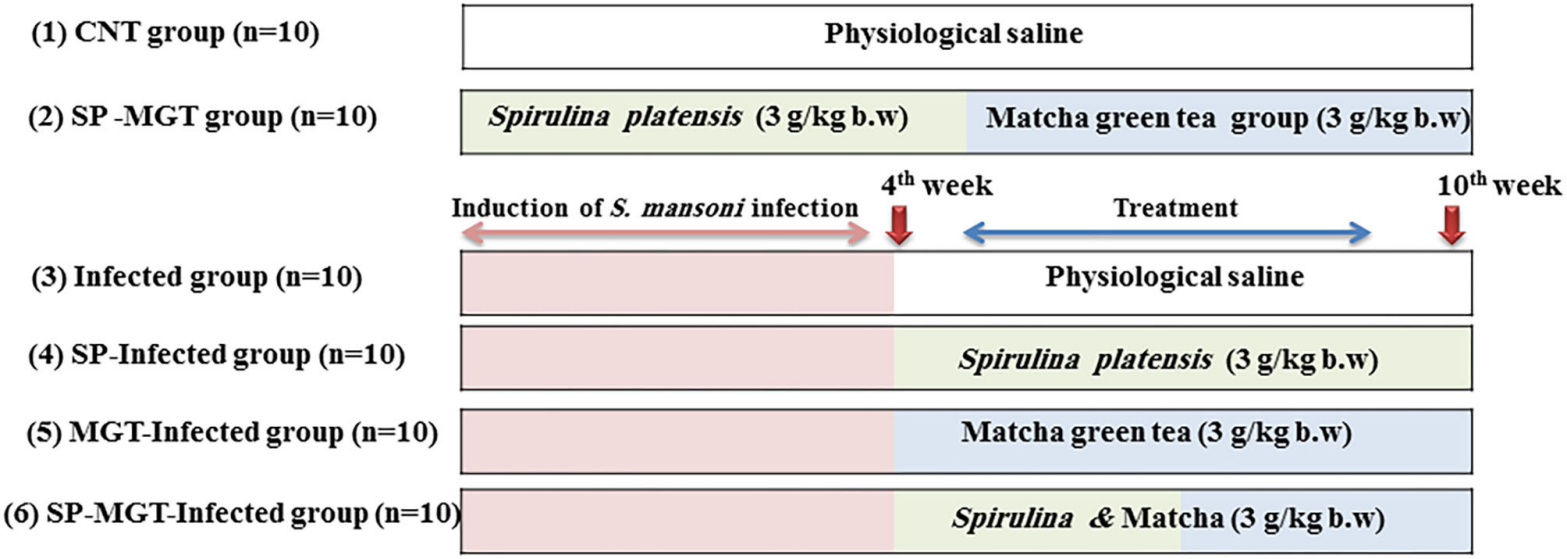
Time scheme, treatment protocol, and doses of used SP and MGT.

As presented in Figure 1, the animals were randomly allocated into the following six experimental groups (*n* = 10 per group):

1. Control group (CNT): mice received oral physiological saline every day and kept without infection.
2. SP–MGT group: mice were treated with 3 g/kg bodyweight of SP (30) suspended in sterile physiological saline (100 ml) plus 3 g/kg bodyweight/day of MGT (31) suspended in distilled water using a gastrostomy tube.
3. *S. mansoni* group (infected): mice were infected with *S. mansoni* cercariae (100 ± 10/mouse) using the tail immersion method.
4. SP-infected group: mice were infected with S. *mansoni* and treated orally with an SP suspension at the same dose as mentioned earlier.
5. MGT-infected group: mice were infected with S. *mansoni* and treated orally with MGT suspension as described earlier.
6. SP–MGT-infected group: mice were treated with a combination of SP and MGT after *S. mansoni* infection in the same manner and same doses as described earlier.

The treatment with SP and MGT started 4 weeks after *S. mansoni* infection and continued until the 10th week. Blood samples were collected from the retro-orbital venous sinus in plain test tubes 24 h after the last treatment. The samples were left to clot for approximately 15 min and then centrifuged at 3,000 rpm (4°C) to obtain the serum. The separated serum samples were kept at −80°C for further analysis. The mice were killed, and the liver and spleen tissues were collected and preserved in 10% neutral buffered formalin for histopathological assessment.

### Biochemical Analysis

Serum alanine and aspartate aminotransferases (ALT and AST, respectively) activities (Randox, UK), as well as the total protein and albumin levels (Stanbio Laboratory, USA), were measured using the Photometer 5010V5+ (BM Co, Germany) following the standard protocols.

### Oxidative Stress/Antioxidant Marker Analysis

Malondialdehyde (MDA), total antioxidant capacity (TAC), catalase (CAT), superoxide dismutase (SOD), and glutathione peroxidase (GSH-Px) were measured in serum samples using commercial test kits obtained from Bio-Chain Inc., USA.

### Cytokine Analysis

The serum levels of tumor necrosis factor-alpha (TNF-α), interferon-gamma (INF-γ), and interleukins (IL-10 and IL-13) were evaluated using commercial ready-made ELISA Kits (Quantikine Co., USA).

### Histopathological Examination

After storing the hepatic and splenic tissues in 10% formalin, the tissues were removed and immersed in serial ascending dilutions of ethanol. The tissue specimens were then dipped in paraffin to obtain the paraffin blocks. The blocks were cut into 5-μm-thick sections. Subsequently, the sections were stained with hematoxylin and eosin, as previously described by Bancroft and Layton (80).

### Statistical Analysis

Data were expressed as mean ± standard error of the mean (SEM). The differences among the biochemical parameters, antioxidant/oxidative stress, and inflammatory biomarkers were analyzed via one-way analysis of variance, followed by Duncan's comparisons tests. All statistical analyses were conducted using the SPSS software version 20 (version 20, USA). Differences were considered statistically significant at *P* < 0.05.

## Results

### Serum Hepatic Injury Biomarkers

The effect of SP and/or MGT on liver activity was evaluated by measuring the serum levels of ALT, AST, total (T) protein, albumin, and globulins in *S. mansoni*-infected mice and shown in Table 1. *S. mansoni* infection (infected group) exhibited elevation of serum ALT and AST and reduction of the total protein, albumin, and globulin levels compared with the control group (*P* < 0.05). Contrarily, the concomitant administration of SP and/or MGT significantly reduced the hepatic damage as demonstrated in the SP-infected, MGT-infected, and SP–MGT-infected groups relative to the infected non-treated group (*P* < 0.05). Treatment with the combination of SP and MGT (SP–MGT-infected group) significantly restored serum total protein and globulin to concentrations similar to those of the uninfected controls (*P* < 0.05).

**Table 1:** Serum liver biomarkers in response to co-treatment with *Spirulina platensis* and/or matcha green tea in *S. mansoni* infected mice.

**Groups**	**Total protein**	**Albumin**	**Globulin**	**ALT**	**AST**
CNT	7.40 ± 0.30	3.20 ± 0.10	3.90 ± 0.26	36.14 ± 1.60	26.62 ± 0.77
SP–MGT	7.83 ± 0.20	3.32 ± 0.12	4.51 ± 0.27	36.64 ± 1.42	27.09 ± 1.86
Infected	1.84 ± 0.27^#^	1.17 ± 0.16^#^	0.87 ± 0.23^#^	98.31 ± 1.78^#^	86.48 ± 2.23^#^
SP-Infected	3.27 ± 0.15^#$^	2.01 ± 0.19^#$^	1.62 ± 0.09^#$^	55.31 ± 0.56^#$^	56.12 ± 1.07^#$^
MGT-Infected	4.41 ± 0.18^#$^	1.75 ± 0.27^#$^	2.83 ± 0.10^#$^	59.76 ± 1.36^#$^	53.35 ± 0.94^#$^
SP–MGT-Infected	6.59 ± 0.41^$^	2.46 ± 0.21^#$^	3.34 ± 0.12^$^	33.56 ± 1.13^$^	32.99 ± 1.60^#$^

*All data were expressed as mean ± SEM (n = 10/group)*.

*^#^ or ^$^ refers to the statistical significance at P < 0.05 against the control and S. mansoni (infected) groups, respectively. ALT, alanine aminotransferase; AST, aspartate aminotransferase*.

### Oxidative Stress and Antioxidant Markers

**Figure 2:**
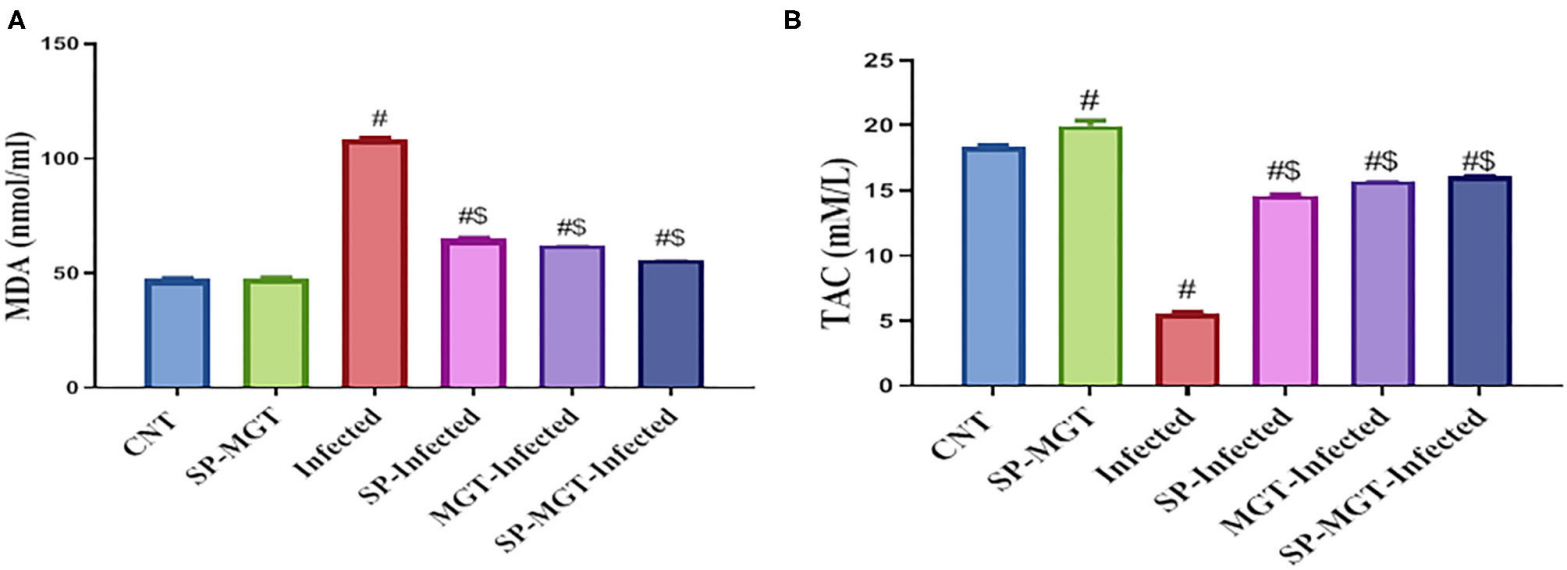
Malondialdehyde (MDA; **A**), and total antioxidant capacity (TAC; **B**) levels in serum of mice in response to co-treatment with *Spirulina platensis* and/or matcha green tea in *S. mansoni* infected mice. Data were displayed as mean ± SEM (*n* = 10/group). ^#^ or ^$^ refers to the statistical significance at *P* < 0.05 against control and *S. mansoni* (infected) groups, respectively.

**Figure 3:**
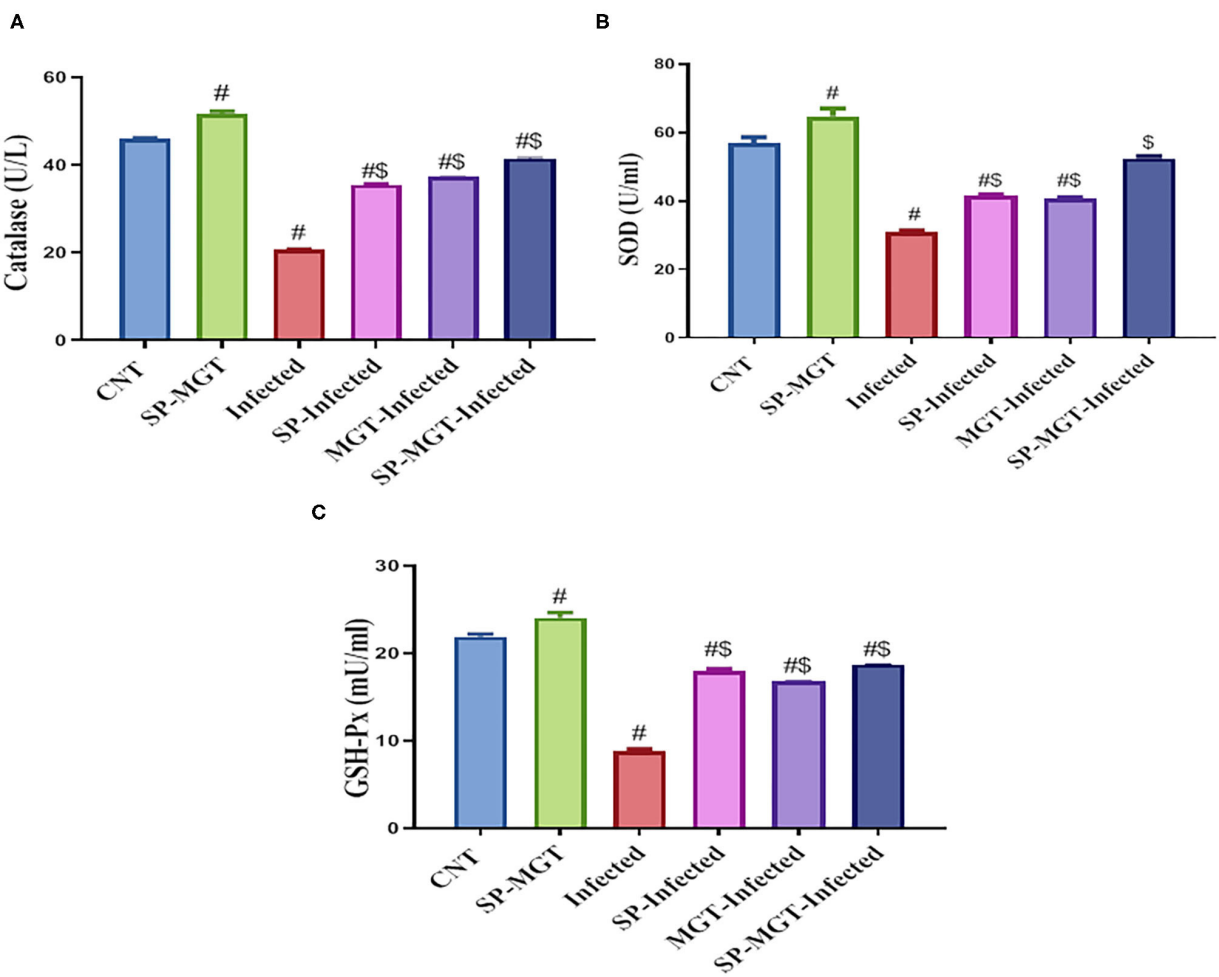
Catalase (CAT; **A**), superoxide dismutase (SOD; **B**), and glutathione peroxidase (GSH-Px; **C**) activities in serum of mice in response to co-treatment with *Spirulina platensis* and/or matcha green tea in *S. mansoni* infected mice. Data were displayed as mean ± SD (*n* = 10/group). ^#^ or ^$^ refers to the statistical significance at *P* < 0.05 against control and *S. mansoni* (infected) groups, respectively.

The antioxidant effects of SP and/or MGT against *S. mansoni*-mediated oxidative stress are presented in Figures 2, 3. Serum MDA was significantly elevated, and the CAT, SOD, and GSH-Px activities and TAC levels were significantly reduced in infected untreated mice compared with the control group. The treatment of *S. mansoni-*infected mice with SP and/or MGT significantly attenuated the changes in MDA, TAC, CAT, SOD, and GSH-Px activities (*P* < 0.05 vs. infected). In addition, SP–MGT-treated mice had higher activities of CAT, SOD, GSH-Px, and TAC compared with the control mice (*P* < 0.05).

### Serum Cytokines

**Figure 4:**
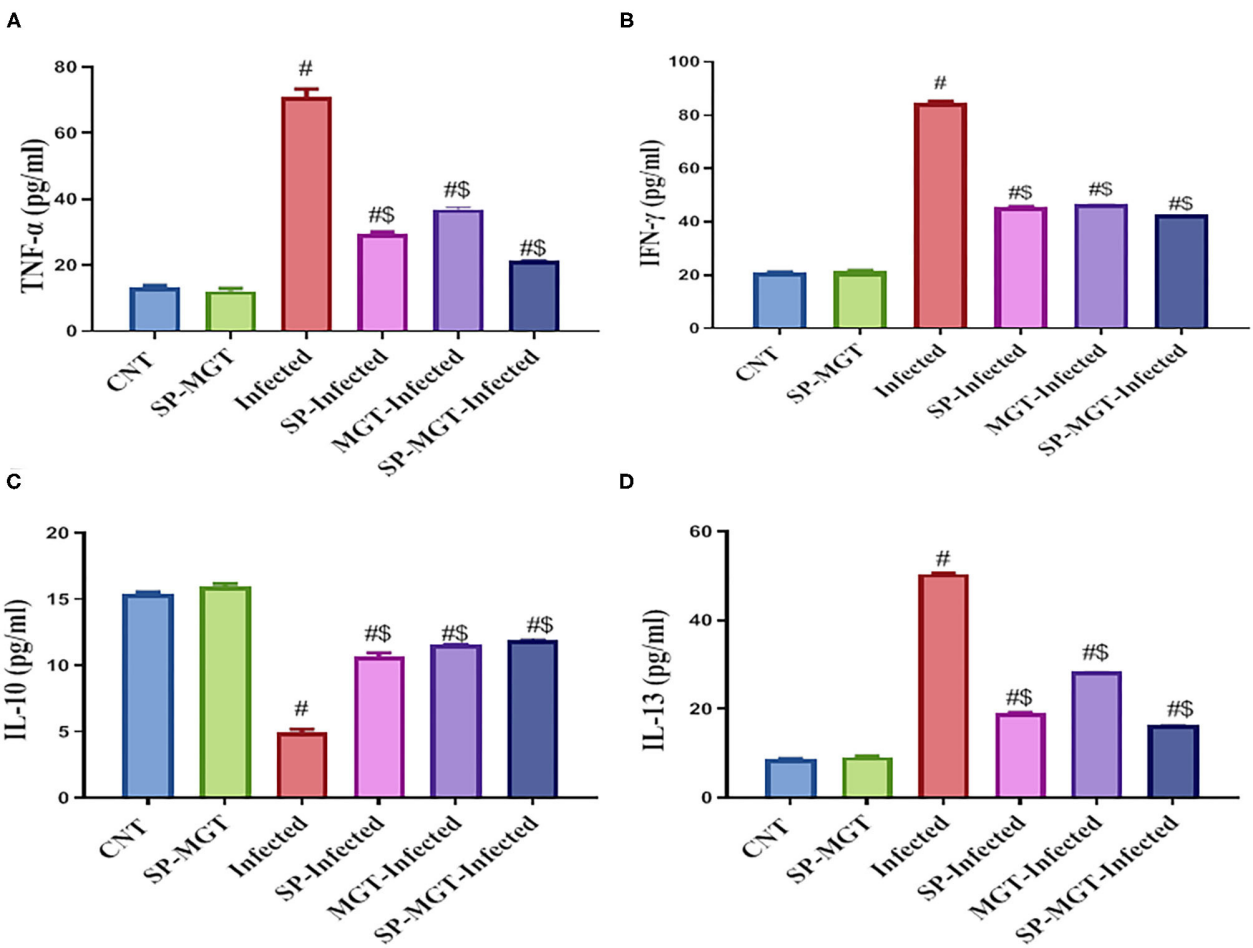
Tumor necrosis factor-alpha (TNF-α; **A**), interferon-gamma (IFN-γ; **B**) levels, interleukin 10 (IL-10; **C**), and interleukin-13 (IL-13; **D**) in serum of mice in response to co-treatment with *Spirulina platensis* and/or matcha green tea in *S. mansoni* infected mice. Data were displayed as mean ± SEM (*n* = 10/group). ^#^ or ^$^ refers to the statistical significance at *P* < 0.05 against control and *S. mansoni* (infected) groups, respectively.

The serum levels of TNF-α, IFN-γ, and IL-13 were significantly elevated, whereas that of IL-10 significantly reduced in *S. mansoni*-infected mice compared with the serum levels in the uninfected controls (Figure 4) (*P* < 0.05). Treatment with SP and/or MGT attenuated the changes in serum cytokines induced by *S. mansoni* infection; TNF-α, IFN-γ, and IL-13 were reduced, and IL-10 was significantly increased in SP- and/or MGT-treated infected mice compared with untreated infected mice (Figure 4) (*P* < 0.05).

### Histopathological Assessments

**Figure 5:**
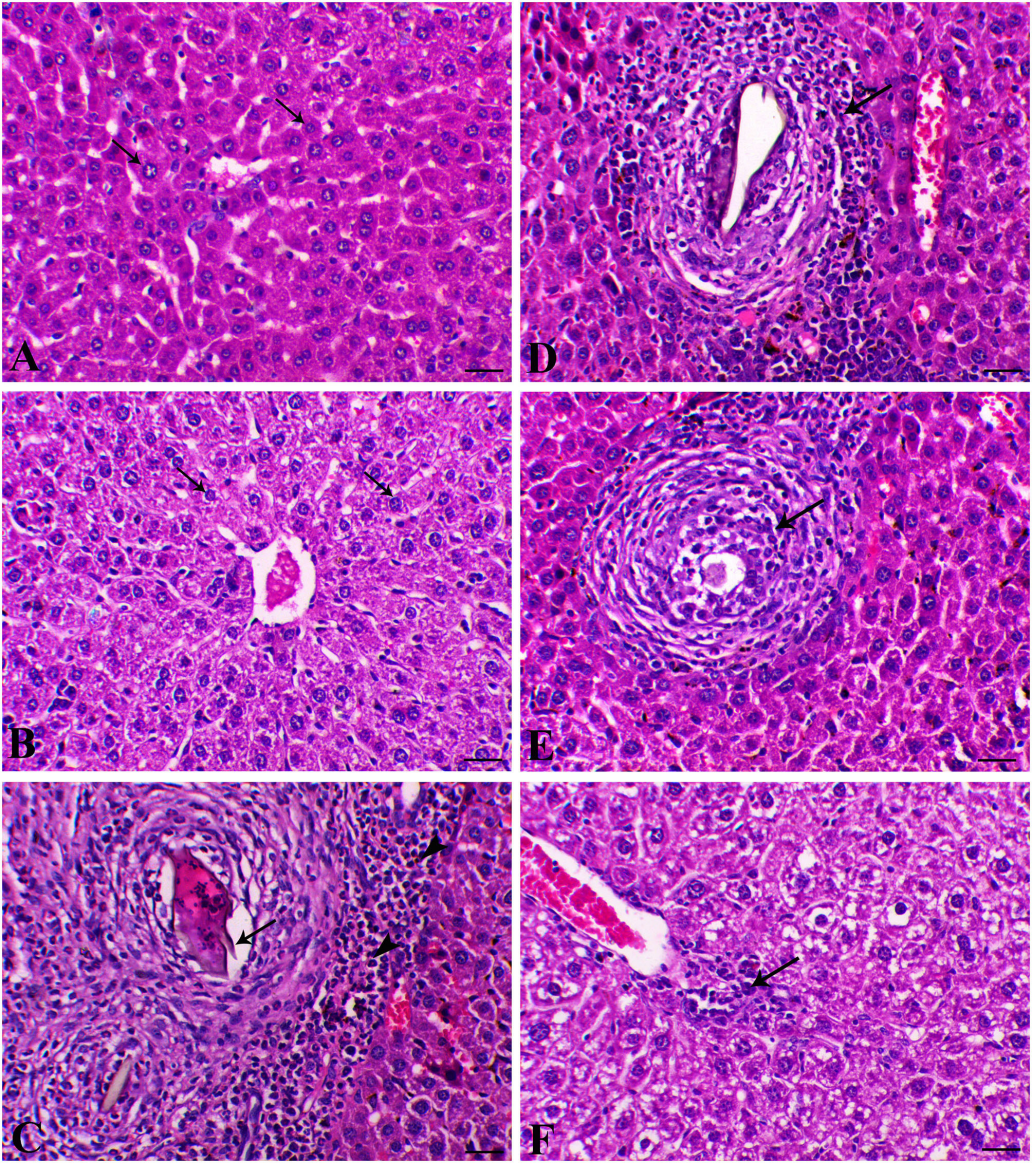
Hepatic histological sections of control group **(A)** (arrows indicate normal polygonal hepatic cell with central vesicular nucleus), SP–MGT **(B)** (arrows indicate normal hepatocytes), Infected group **(C)** (arrow indicates the schistosome egg, which revealing lateral spine, arrowheads indicates eosinophils), SP-infected group **(D)** (arrow indicates small-sized granuloma), MTG-infected group **(E)** (arrow indicates small-sized granuloma), and SP + MGT-infected group **(F)** (arrow indicates mild lymphocytic cells infiltration).

**Figure 6:**
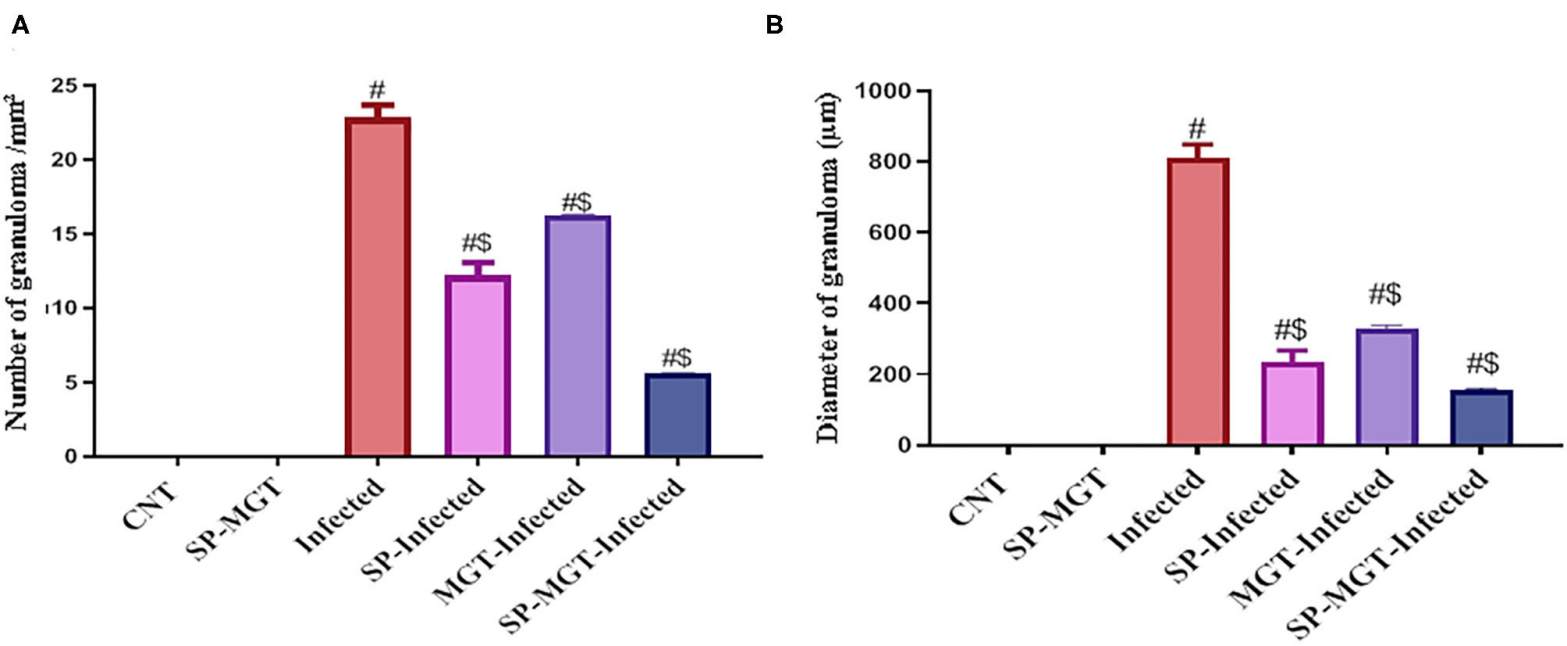
Quantitative scoring of number **(A)** and diameter **(B)** of granuloma in hepatic tissues. Data were displayed as mean ± SEM (*n* = 5/group). ^#^ or ^$^ refers to the statistical significance at *P* < 0.05 against control and *S. mansoni* (infected) groups, respectively.

The liver sections are presented in Figure 5. Control animals exhibited normal hepatocytes radiating in cords around the central vein. The hepatic tissues of animals treated with SP or MGT were normal. *Schistosoma*-infected animals exhibited numerous granulomatous lesions around the parasites and their eggs in the liver. The lesions were usually associated with marked inflammation and fibrosis, including infiltration of lymphocytes, macrophages, and large numbers of eosinophils. The number of granulomas decreased in infected animals treated with MGT compared with the number of lesions in infected untreated animals (Figure 6). Animals treated with SP exhibited smaller granulomas, mostly around the disintegrated parasite. Conversely, infected animals treated with both SP and MGT had fewer and smaller granulomas and mild periportal lymphocytic cell infiltration compared with untreated infected mice (*P* > 0.01). Interestingly, the combination group exhibited a significant decrease in these parameters in comparison with a single treatment of SP or MGT (*P* > 0.01).

**Figure 7:**
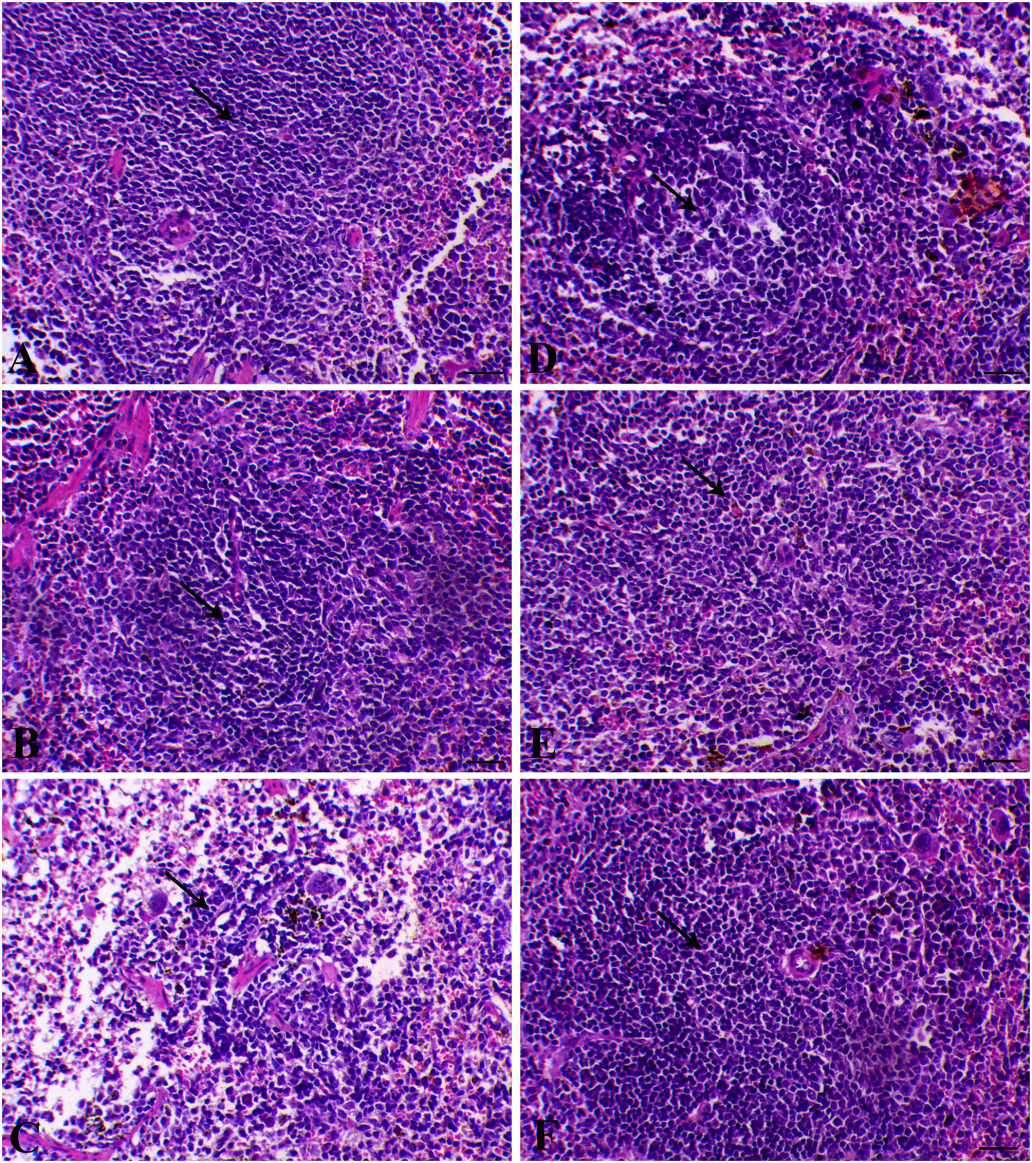
Splenic histological sections of control group **(A)** (arrow indicates normal lymphoid follicle), SP–MGT group **(B)** (arrow indicates normal lymphoid follicle), Infected group **(C)** (arrow indicates severe degree of lymphoid depletion), SP-infected group **(D)** (arrow indicates plenty of lymphoid cells), MTG-infected group **(E)** (arrow indicates an increase of lymphoid elements), and SP + MGT-infected group **(F)** (arrow indicates normal lymphoid pulp).

The splenic sections are presented in Figure 7. Control animals exhibited normal red and white pulps. Animals treated with only SP or MGT exhibited normal lymphoid cells around the central arteriole. Red pulp congestion associated with hemosiderin pigment deposition was observed in infected animals. In addition, a marked degree of lymphoid depletion accompanied by the appearance of reticular fibers was observed in the white pulp of infected mice. *Schistosoma*-infected mice treated with MGT exhibited marked decreases in lymphoid necrosis with lymphoid cell proliferation. Restoration of lymphoid follicles occurred in infected animals treated with SP, whereas in infected animals treated with both SP and MGT, the lymphoid follicles were normal.

## Discussion

Schistosomiasis is one of the most widespread parasitic sicknesses. In addition to the high economic costs, schistosomiasis negatively impacts the well-being of the people in subtropical and tropical countries (32). In the last few decades, researchers have aimed to develop effective antischistosomal drugs with few adverse effects (33). However, this objective seems to be out of reach. Green tea is a widely consumed drink worldwide. It contains several active compounds, including polyphenols (tea catechins), methylxanthines, and essential oils (34). Previous reports have demonstrated the antiparasitic and biological activities of green tea (23, 35, 36). In this report, we demonstrated the antischistosomal activity of SP and MGT through their hepatosplenic antioxidant and anti-inflammatory protective effects.

*S. mansoni* infection causes hepatocellular injury and increases the circulating levels of hepatic enzymes, such as ALT and AST. In addition, it impairs protein synthesis (37). In our mouse model, the circulating levels of AST and ALT were significantly increased, whereas the total protein, albumin, and globulins were decreased after *S. mansoni* infection. Other investigators also illustrated hepatic damage mediated by *S. mansoni* infection (elevated ALT, AST, and ALP levels) in animal models (38–40). Liver damage also occurs, as indicated by the presence of inflammatory granulomas resulting from egg deposition as well as worms and their toxins (41). *S. mansoni* infection also leads to the reduction of the levels of total protein, albumin, and globulin (42–44). Moreover, hypoalbuminemia is associated with collagen deposition (40). Serum protein and albumin levels may also decrease with *S. mansoni* infection due to malabsorption after the extrusion of large egg numbers and intestinal mucosal damage or decreased synthesis due to hepatic cell injury (45). In our study, the serum biochemical data correlated with the histopathological results, and both confirmed liver damage.

Liver damage was resolved by the administration of SP and MGT, especially the combined treatment. Mohamed et al. (66) revealed that serum ALT, AST, and ALP activities were significantly reduced after treatment of *S. mansoni*-infected mice with blue-green algae, which is consistent with our results. The SP administration also significantly attenuated the elevated serum AST and ALT after doxorubicin treatment in rats; the presence of β-carotene in SP correlated with the decline in cell damage, particularly DNA damage (46). Al-Qahtani and Binobead (78) attributed the protective effects of SP against D-galactosamine-induced liver damage to the phenolic compounds in SP. SP can prevent hepatocyte deformations, inhibit hepatic enzymes, and prevent further cell damage. Numerous studies also revealed the protective potency of green tea toward various poisons and medicines, such as malathion (25), leflunomide (47), and micromycin (48). Our results were similar to the results of Dobrzynska et al. (81), Augustyniak et al. (79), El-Beshbishy et al. (60), and Miranda et al. (83). In these publications, green tea extracts lowered serum liver enzymes and protected liver cells from damage. In addition, co-administration of green tea ameliorated *S. mansoni*-induced liver damage in mice, as demonstrated by the significant decreases in ALT levels, the restoration of hepatocyte structure, and the decrease in perivascular collagen fibers (49).

A dynamic physiological connection exists between parasitic diseases and the antioxidant defense mechanisms of the infected host (50). Highly significant increases in hepatic lipid peroxides and glutathione depletion were found to occur in *S. mansoni*-infected mice (51). Interestingly, oxidative stress due to schistosomiasis increased the level of MDA (39) and reduced levels of SOD, CAT, and GSH-Px (41). Jatsa et al. (82) also demonstrated increased liver MDA concentration, with significant inhibition of glutathione concentration and CAT activity after schistosomiasis. The increased MDA levels might be due to the liberation of a huge amount of superoxide radicals by macrophages present in hepatic granulomas during *S. mansoni* infection. In addition, CAT is an endogenous antioxidant enzyme, and its depletion indicates an increase in the number of free radicals, inducing cellular damage (52). The cellular antioxidant system disturbances in schistosomiasis might arise in response to the stimulation of the host immune system, leading to the release of oxygen-derived free radicals as a premier non-specific defense response toward parasitic infection (53). Oxidative injury after *S. mansoni* infection was previously described, including increased MDA and NO levels and decreased GSH, CAT, and SOD activities (49, 54, 55). Our work confirmed the oxidative damage induced by schistosomiasis, which is indicated by significantly elevated serum levels of MDA and reduced CAT, SOD, and GSH-Px activities as well as TAC levels in *S. mansoni*-infected mice. The oxidative damage was reversed by SP and MGT supplementation.

The potential antioxidant activity of SP may be linked to its constituents, including chlorophyll, carotene, phenolic compounds, selenium, gamma-linolenic acids, and tocopherol. These constituents have the ability to protect against free radical-induced cellular transformation (56, 57). Another active component of SP is C-phycocyanin, which exhibited superoxide and hydroxyl radical scavenging activity and inhibition of lipid peroxidation (58). Moreover, pretreatment with SP reduces the toxic effects of cadmium and doxorubicin, as indicated by reduced MDA and NO levels and increased GSH and SOD levels in the liver tissue (46, 59). Among the major constituents of green tea are polyphenols, especially catechins, which prevent lipid peroxidation and have strong superoxide, hydrogen peroxide, and nitric acid scavenging activity (25, 60). Another active component of matcha is theanine, which can be converted to glutamine (29). Glutamate can alleviate mitochondrial damage, rejuvenate intermediate metabolites of the TCA cycle, and increase ATP production via the oxidative phosphorylation process to quench oxidative damage (61). Our findings are in agreement with a previous report demonstrating the role of green tea in quenching oxidative stress mediated by *S. mansoni* through the elevation of the TAC, SOD, and CAT activities in the liver and reduction of hepatic lipid peroxidation (49).

Of note, cytokines are critical in the pathogenesis of *S. mansoni* infection and are responsible for the extent of fibrosis and granuloma formation (62). The key mediator cytokine of liver fibrosis in *S. mansoni* infections is IL-13 (63). TNF-α participates in the maintenance of the granulomatous response (64). IFN-γ elicits free radical production, M1 macrophage expansion, and apoptosis (85). In addition, IL-10 plays an important role in the modulation of cytokine networks in schistosomiasis. The association between hepatic granuloma and fibrosis after *S. japonicum* infection and other mediators (IL-13 and tissue transglutaminases) has been reported (65). Silveira-Lemos et al. (84) also revealed that TNF-α and IL-10 modulate the development of granuloma in both humans and experimental schistosomiasis models. In line with these investigations, our study demonstrated that *S. mansoni* parasitic infection mediated the production of inflammatory fibrogenic factors via the elevation of serum TNF-α, IFN-γ, and IL-13 levels coupled with reduced IL-10 levels. Blue-green algae significantly reduced the serum levels of TNF-α in *S. mansoni-*infected mice (66). The anti-inflammatory effects of *S. platensis* were attributed to the inhibition of the NF-κB pathway and the subsequent suppression of pro-inflammatory cytokine production (67). Furthermore, phycocyanin, an active SP component, effectively limits the cyclooxygenase-2 inflammatory pathway and myeloperoxidase activity, scavenges free radicals, and inhibits lipid peroxidation (68). In this study, the inflammatory cytokines (TNF-α, IFN-γ, IL-10, and IL-13) returned to control serum levels after SP and/or MGT treatment of *S. mansoni-*infected mice, demonstrating the anti-inflammatory effects of SP and MGT. Green tea and green tea flower extracts downregulate the expression of inflammation markers, including IL-β1, IL-6, and TNFα, in human gingival epithelial keratinocytes and mouse liver treated with lipopolysaccharides (69, 70). Theanine, the main component of MGT, and its metabolites, such as glutamine and ethyleneimine, alleviate inflammation by reducing NF-κB activation and neutrophil accumulation in tissues and dampening the Ca^2+^ channels to block inflammatory mediator release (71).

Schistosomiasis causes the formation of hepatic granulomas and fibrosis, in addition to necrotic changes in the liver (33). In agreement with previous studies, our experiments revealed that chronic inflammation is among the pathological lineaments of *S. mansoni*, causing intense fibrosis in the infected tissues and organs (72). An increased number and diameter of granulomas and extensive fibrous tissue accumulation were detected in response to *S. mansoni* in several previous works (55, 73). In agreement with previous studies (74, 75), we detected parasitic egg granulomas with an increased spleen size after *S. mansoni* infection. SP has no harmful effects or organ toxicity; however, it remarkably reduced the incidence of liver tumors, indicating its prospective therapeutic effects on our model (56, 57). Surprisingly, green tea treatment of mice infected with *S. mansoni* caused an obvious refinement of most hepatocytes surrounding the blood vessels and reduced perivascular collagenous fibers, whereas some hepatocytes still exhibited cytoplasmic degeneration (49). The spleen and liver are connected via the portal vein system; thus, the spleen may contribute to the development of liver fibrosis through the infiltration of monocytes and cytokine production. As presented in our results, *S. mansoni* infection resulted in a series of histopathological changes in the spleen indicated by marked lymphoid necrosis within the white pulp associated with the infiltration of macrophages and histiocytes. Interestingly, treatment of mice with a combination of SP and MGT restored the normal histology of the spleen and lymphoid follicles. These changes are consistent with several previous reports demonstrating the antioxidant roles of SP and MGT in the restoration of lymphoid follicles and splenic pulps via altered inflammatory gene expression in the splenocytes (76, 77).

## Conclusion

Taken together, the present findings emphasize that *S. mansoni* infection induces hepatosplenic injury in mice. Combined treatment with SP and MGT is a new favorable natural approach for minimizing the pathological alterations in the liver and spleen after *S. mansoni* infection, as indicated by restored antioxidant status, reduced inflammatory markers, and normal tissue architecture. Future investigations should consider the pharmacological potentials of SP and MGT, as well as other transduction pathways involved in their antischistosomal activity.

## Data Availability Statement

The original contributions presented in the study are included in the article/supplementary material, further inquiries can be directed to the corresponding author/s.

## Ethics Statement

The animal study was reviewed and approved by the ethical approval and the protocol of this experiment were authorized by the Animal Ethical Committee of the Faculty of Veterinary Medicine, Kafrelsheikh University, Egypt, which complies with all relevant Egyptian legislations in publication and research. The ethical approval number is KFS-2019/2.

## Author Contributions

AR, EE, GE, and DE-S were involved in the conception of the research idea and participated in methodology design, supervision, and data analysis and interpretation. EE, GE, AS, SM, AA-B, and WA participated in methodology and data analysis. AR, EE, GE, WA, and DE-S drafted and prepared the manuscript for publication and revision. All authors contributed to the article and approved the submitted version.

## Conflict of Interest

The authors declare that the research was conducted in the absence of any commercial or financial relationships that could be construed as a potential conflict of interest.

## Abbreviations

SP: *Spirulina platensis*
MGT: matcha green tea
AST: aspartate transferase
ALT: alanine transferase
MDA: malondialdehyde
CAT: catalase
GSH-Px: glutathione peroxidase
SOD: superoxide dismutase
TAC: total antioxidant capacity
TNF-α: tumor necrosis factor
IFN-γ: interferon-gamma
IL-10: interleukin-6
IL-13: interleukin-13.
